# Supplementary material for: Attitudes, beliefs and behaviors of religiosity, spirituality, and cultural competence in the medical profession: A cross-sectional survey study
Source: PLoS One. 2021 Jun 15;16(6):e0252750. doi: 10.1371/journal.pone.0252750 (PMC8205176; doi:10.1371/journal.pone.0252750)
Supplement: S1 Appendix — (PDF) [file pone.0252750.s001.pdf]

# Attitudes, Beliefs and Behaviors of Religiosity, Spirituality, and Cultural Competence in the Medical Profession

Please complete the survey below.

Thank you!

---

Your participation in this survey is completely voluntary. If you wish to exit this survey prior to completion you may exit it at any time.

This survey is designed to gather information to help investigate the relationships between religion, spirituality, and cultural competence.

This survey should take approximately 10-15 minutes to complete. Please answer each of the following questions to the best of your ability. If you are not sure of an answer to a question, please provide your best estimate. Your responses will be kept confidential. This research was approved by the Institutional Review Board at the University of South Carolina #PROxxxx--. If you have any questions or concerns, please contact the Principal Investigator, Ann Blair Kennedy at kenneda5@greenvillemed.sc.edu, or the Institutional Review Board, at 803-576-7276.

---

Are you willing to take this survey?

◯ Yes
◯ No

---

Are you a resident, fellow, or physician OR a medical student involved in clinical patient care?

◯ Yes
◯ No

---

Are you currently attending medical school or practicing medicine in the United States?

◯ Yes
◯ No

*This survey has been adapted with permission from the Clinical Cultural Competency Questionnaire (CCCQ) developed by Robert C. Like, MD, MS, Professor and Director of the Center for Healthy Families and Cultural Diversity, Department of Family Medicine and Community Health, Rutgers Robert Wood Johnson Medical School. The CCCQ was used in a project entitled, "Assessing the Impact of Cultural Competency Training Using Participatory Quality Improvement Methods," funded by the Aetna Foundation (http://rwjms.rutgers.edu/departments_institutes/family_medicine/chfcd/grants_projects/aetna.html). Any results obtained in future projects making use of the CCCQ are solely the responsibility of project investigators and do not necessarily represent the official views of the Aetna Foundation or its affiliates.*

**We would like to start by gathering some information about you.**

Position

- ◯ Medical Student
- ◯ Resident
- ◯ Fellow
- ◯ Attending Physician

State of current practice/medical school?

- ◯ AL
- ◯ AK
- ◯ AZ
- ◯ AR
- ◯ CA
- ◯ CO
- ◯ CT
- ◯ DE
- ◯ FL
- ◯ GA
- ◯ HI
- ◯ ID
- ◯ IL
- ◯ IN
- ◯ IA
- ◯ KS
- ◯ KY
- ◯ LA
- ◯ ME
- ◯ MD
- ◯ MA
- ◯ MI
- ◯ MN
- ◯ MS
- ◯ MO
- ◯ MT
- ◯ NE
- ◯ NV
- ◯ NH
- ◯ NJ
- ◯ NM
- ◯ NY
- ◯ NC
- ◯ ND
- ◯ OH
- ◯ OK
- ◯ OR
- ◯ PA
- ◯ RI
- ◯ SC
- ◯ SD
- ◯ TN
- ◯ TX
- ◯ UT
- ◯ VT
- ◯ VA
- ◯ WA
- ◯ WV
- ◯ WI
- ◯ WY
- ◯ Washington DC

Location Type of Medical School/Current Practice

☐ Urban ☐ Suburban
☐ Rural ☐ Tribal

| | |
|---|---|
| Primary Specialty (intended or actual) | ◯ Allergy & Immunology<br>◯ Anesthesiology<br>◯ Dermatology<br>◯ Emergency Medicine<br>◯ Family Medicine<br>◯ General Surgery<br>◯ Internal Medicine<br>◯ Internal Medicine-Pediatrics<br>◯ Interventional Radiology<br>◯ Medical Genetics<br>◯ Neurology<br>◯ Neurosurgery<br>◯ Obstetrics & Gynecology<br>◯ Ophthalmology<br>◯ Orthopedic Surgery<br>◯ Otolaryngology<br>◯ Pediatrics<br>◯ Physical Medicine & Rehabilitation<br>◯ Preventive Medicine<br>◯ Psychiatry<br>◯ Radiology<br>◯ Pathology<br>◯ Urology<br>◯ Other<br>◯ None or Undecided |
| Please describe your other specialty | ______________________________ |
| Race/Ethnicity (choose all that apply) | ☐ American Indian or Alaska Native<br>☐ Asian<br>☐ Black or African American<br>☐ Hispanic or Latino<br>☐ Native Hawaiian or Pacific Islander<br>☐ White<br>☐ Other<br>☐ Decline to Answer |
| Please describe other race/ethnicity | ______________________________ |
| Gender Identity | ☐ Female<br>☐ Male<br>☐ Agender<br>☐ Gender Nonbinary<br>☐ Transgender Male<br>☐ Transgender Female<br>☐ Genderqueer<br>☐ Bigender<br>☐ Other<br>☐ Decline to Answer |
| Please describe other gender identity | ______________________________ |
| Age | ________________________<br>(Round to the nearest year) |

| | |
|---|---|
| Religious Identity | ☐ Agnostic<br>☐ Atheist<br>☐ Baha'i<br>☐ Buddhist<br>☐ Catholic<br>☐ Hindu<br>☐ Jewish<br>☐ Mormon<br>☐ Muslim<br>☐ Protestant<br>☐ Sikhism<br>☐ Other<br>☐ Decline to Answer |
| Please describe other religious identity | __________________________________ |

**We are also interested in your patient population.**

**How would you describe your patient population by ethnicity and race? (Please estimate to the best of your ability if you do not have exact data readily available.)**

| | < 1% | 1-10% | 10-25% | 25-50% | 50-75% | 75-90% | 90-99% | >99% | Do not know |
|---|---|---|---|---|---|---|---|---|---|
| American Indian or Alaska Native | ○ | ○ | ○ | ○ | ○ | ○ | ○ | ○ | ○ |
| Asian | ○ | ○ | ○ | ○ | ○ | ○ | ○ | ○ | ○ |
| Black or African American | ○ | ○ | ○ | ○ | ○ | ○ | ○ | ○ | ○ |
| Hispanic or Latino | ○ | ○ | ○ | ○ | ○ | ○ | ○ | ○ | ○ |
| Native Hawaiian or Pacific Islander | ○ | ○ | ○ | ○ | ○ | ○ | ○ | ○ | ○ |
| White | ○ | ○ | ○ | ○ | ○ | ○ | ○ | ○ | ○ |
| Other | ○ | ○ | ○ | ○ | ○ | ○ | ○ | ○ | ○ |

Describe "other" race ________________________________

**How would you describe your patient population by religion? (Please estimate to the best of your ability if you do not have exact data readily available.)**

| | < 1% | 1-10% | 10-25% | 25-50% | 50-75% | 75-90% | 90-99% | >99% | Do not know |
|---|---|---|---|---|---|---|---|---|---|
| Agnostic | ○ | ○ | ○ | ○ | ○ | ○ | ○ | ○ | ○ |
| Atheist | ○ | ○ | ○ | ○ | ○ | ○ | ○ | ○ | ○ |
| Baha'i | ○ | ○ | ○ | ○ | ○ | ○ | ○ | ○ | ○ |
| Buddhist | ○ | ○ | ○ | ○ | ○ | ○ | ○ | ○ | ○ |
| Catholic | ○ | ○ | ○ | ○ | ○ | ○ | ○ | ○ | ○ |
| Hindu | ○ | ○ | ○ | ○ | ○ | ○ | ○ | ○ | ○ |
| Jewish | ○ | ○ | ○ | ○ | ○ | ○ | ○ | ○ | ○ |
| Mormon | ○ | ○ | ○ | ○ | ○ | ○ | ○ | ○ | ○ |
| Muslim | ○ | ○ | ○ | ○ | ○ | ○ | ○ | ○ | ○ |
| Protestant | ○ | ○ | ○ | ○ | ○ | ○ | ○ | ○ | ○ |
| Sikhism | ○ | ○ | ○ | ○ | ○ | ○ | ○ | ○ | ○ |
| Other | ○ | ○ | ○ | ○ | ○ | ○ | ○ | ○ | ○ |
| I do not identify with a particular religion | ○ | ○ | ○ | ○ | ○ | ○ | ○ | ○ | ○ |

Describe "other" religion

__________________________________

**Next, we are interested in your personal values and beliefs around religion and spirituality. For the purposes of this survey, these terms will be defined as follows:**
**1) Religion - "The belief in and worship of a superhuman controlling power, especially a personal God or gods."**
**2) Religiosity - "Strong religious feeling or belief."**
**3) Spirituality - "The quality of being concerned with the human spirit or soul as opposed to material or physical things."**

| | |
|---|---|
| How often do you typically attend religious services? | ◯ At least once a week<br>◯ 1-2 times a month<br>◯ 1-2 times a year<br>◯ Never |
| Approximately how often do you pray? | ◯ Multiple times a day<br>◯ Daily<br>◯ Weekly<br>◯ Monthly<br>◯ Rarely<br>◯ Never |
| How often do you typically practice mindfulness and/or meditate? | ◯ Multiple times a day<br>◯ Daily<br>◯ Weekly<br>◯ Monthly<br>◯ Rarely<br>◯ Never |
| How often do you typically practice meditation with movement (e.g. yoga, Thai Chi, meditative walking, etc.)? | ◯ Multiple times a day<br>◯ Daily<br>◯ Weekly<br>◯ Monthly<br>◯ Rarely<br>◯ Never |
| How important is religion to you in your personal life? | ◯ Very important<br>◯ Somewhat important<br>◯ I don't know<br>◯ Not too important<br>◯ Not at all important |
| How important is spirituality to you in your personal life? | ◯ Very important<br>◯ Somewhat important<br>◯ I don't know<br>◯ Not too important<br>◯ Not at all important |

**In this section, we are interested in how religion and spirituality interact with patient care.**

How often do you ask your patients about their religious or spiritual beliefs?

- ◯ Never
- ◯ Rarely
- ◯ Sometimes
- ◯ Frequently
- ◯ Always

How often do you ask your patients about their cultural beliefs and practices?

- ◯ Never
- ◯ Rarely
- ◯ Sometimes
- ◯ Frequently
- ◯ Always

How often do you refer patients to a chaplain or other service to meet their religious needs?

- ◯ Never
- ◯ Rarely
- ◯ Sometimes
- ◯ Frequently
- ◯ Always

**How knowledgeable are you of the following?**

| | Not at all | A little | Somewhat | Quite a bit | Extremely |
|---|---|---|---|---|---|
| Demographics of diverse racial, and ethnic groups in my community | ○ | ○ | ○ | ○ | ○ |
| Health disparities affecting the populations in my community | ○ | ○ | ○ | ○ | ○ |
| Socioeconomic factors that impact health | ○ | ○ | ○ | ○ | ○ |
| Socioeconomic factors affecting the populations in my community | ○ | ○ | ○ | ○ | ○ |
| Different Healing Traditions (e.g., Ayurvedic Medicine, Traditional Chinese Medicine) | ○ | ○ | ○ | ○ | ○ |
| Historical and contemporary impact of racism, bias, prejudice and discrimination in health care experienced by various population groups in the United States | ○ | ○ | ○ | ○ | ○ |

## How skilled are you in . . .

| | Not at all | A little | Somewhat | Quite | Extremely | I don't know |
|---|---|---|---|---|---|---|
| Eliciting the patient's perspective about health and illness? (e.g., its etiology, name, treatment, course, prognosis) | ◯ | ◯ | ◯ | ◯ | ◯ | ◯ |
| Performing a culturally sensitive physical examination? | ◯ | ◯ | ◯ | ◯ | ◯ | ◯ |
| Providing culturally sensitive patient education, counseling, and treatment plans? | ◯ | ◯ | ◯ | ◯ | ◯ | ◯ |
| Apologizing for cross-cultural misunderstandings or errors? | ◯ | ◯ | ◯ | ◯ | ◯ | ◯ |
| Working with a colleague who makes derogatory remarks about patients from a particular ethnic group? | ◯ | ◯ | ◯ | ◯ | ◯ | ◯ |
| Treating a patient who makes derogatory comments about your racial or ethnic background? | ◯ | ◯ | ◯ | ◯ | ◯ | ◯ |
| Caring for a patient who uses folk healers or alternative therapies | ◯ | ◯ | ◯ | ◯ | ◯ | ◯ |

**How comfortable would you feel the following cross-cultural interactions or situations?**

| | Not at all | A little | Somewhat | Quite | Extremely | I don't know |
|---|---|---|---|---|---|---|
| Caring for patients from culturally diverse backgrounds | ○ | ○ | ○ | ○ | ○ | ○ |
| Caring for patients with limited English proficiency | ○ | ○ | ○ | ○ | ○ | ○ |
| Caring for patients of a different sexual identity than you | ○ | ○ | ○ | ○ | ○ | ○ |
| Caring for patients of a different gender identity than you | ○ | ○ | ○ | ○ | ○ | ○ |
| Interpreting different cultural expressions of pain, distress, and suffering | ○ | ○ | ○ | ○ | ○ | ○ |
| Advising a patient to change behaviors or practices related to cultural beliefs that impair one's health | ○ | ○ | ○ | ○ | ○ | ○ |
| Working with health care professionals from culturally diverse backgrounds | ○ | ○ | ○ | ○ | ○ | ○ |

**Communication**

**How often are the following true for you in your medical practice?**

| | Never | Rarely | Sometimes | Frequently | Always | Not Applicable |
|---|---|---|---|---|---|---|
| Artwork, printed materials, pictures, videos, and health education materials that I use and/or display in my work environment reflect the different cultures and ethnic backgrounds of the clients I serve. | ○ | ○ | ○ | ○ | ○ | ○ |
| I ensure that my patients have access to healthcare services in the language they prefer. | ○ | ○ | ○ | ○ | ○ | ○ |
| I ensure that all notices and communications to individuals and families are written in their language of origin and take into account the average literacy levels of those that I serve. | ○ | ○ | ○ | ○ | ○ | ○ |

**Diversity of Interactions**

**How often are the following true for you in your medical practice?**

| | Never | Rarely | Sometimes | Frequently | Always | I don't know |
|---|---|---|---|---|---|---|
| I advocate for the review of my program's or agency's mission statement, goals, policies, and procedures to ensure that they incorporate principles and practices that promote cultural and linguistic competence. | ○ | ○ | ○ | ○ | ○ | ○ |
| I intervene when I observe other students, staff, or clients within my program or agency engaging in behaviors that show cultural insensitivity, racial biases, and prejudice. | ○ | ○ | ○ | ○ | ○ | ○ |
| I have participated in professional development and training to enhance my knowledge and skills in the provision of services to culturally, and linguistically diverse groups. | ○ | ○ | ○ | ○ | ○ | ○ |

## Importance of Sociocultural Identities

| | Not at all important | Not too important | Somewhat important | Very important |
|---|---|---|---|---|
| How important do you believe sociocultural issues are in your work interactions with patients? | ◯ | ◯ | ◯ | ◯ |
| How important do you believe sociocultural issues are in your work interactions with colleagues? | ◯ | ◯ | ◯ | ◯ |
| How important do you believe sociocultural issues are in your work interactions with other staff? | ◯ | ◯ | ◯ | ◯ |
| How important do you believe it is for health professionals to receive training in cultural diversity and/or multicultural health care? | ◯ | ◯ | ◯ | ◯ |
| How important is religion to health? | ◯ | ◯ | ◯ | ◯ |
| How important is spirituality to health? | ◯ | ◯ | ◯ | ◯ |

How aware are you of your own racial, ethnic, or cultural identity?

◯ Not very aware
◯ Somewhat aware
◯ Very aware
◯ I don't know

How aware are you of your own biases and prejudices against people from certain races, ethnicities, and cultures?

◯ Not very aware
◯ Somewhat aware
◯ Very aware
◯ I don't know

## Training and Education

| | |
|---|---|
| How many hours of training in cultural competence have you had? | ◯ None<br>◯ 1-2 hours<br>◯ 2-5 hours<br>◯ 6-10 hours<br>◯ 10-20 hours<br>◯ >20 hours<br>◯ I don't know |
| Where was the training in cultural competence? | ☑ Medical School<br>☑ Residency<br>☑ Continuing Medical Education<br>☑ Independent/Other |
| Is there anything else you would like to tell us about the intersection of religion and spriritualiity and healthcare and your practice of medicine? | __________________________________ |
