# Supplementary figures and images for: Attitudes, beliefs and behaviors of religiosity, spirituality, and cultural competence in the medical profession: A cross-sectional survey study
Source: PLoS One. 2021 Jun 15;16(6):e0252750. doi: 10.1371/journal.pone.0252750 (PMC8205176; doi:10.1371/journal.pone.0252750)

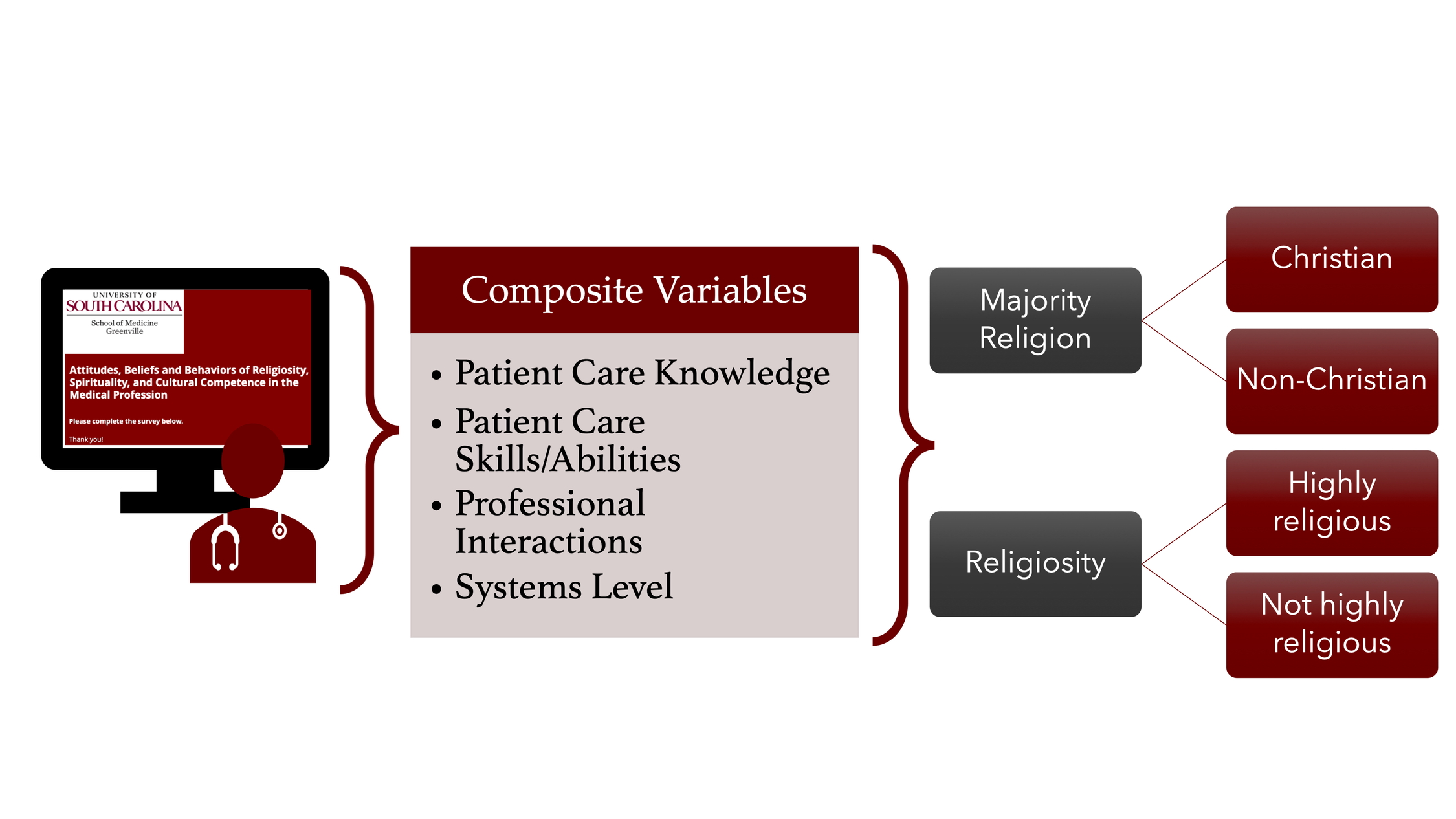

Supplement: S2 Appendix — (TIF) [file pone.0252750.s002.tif]

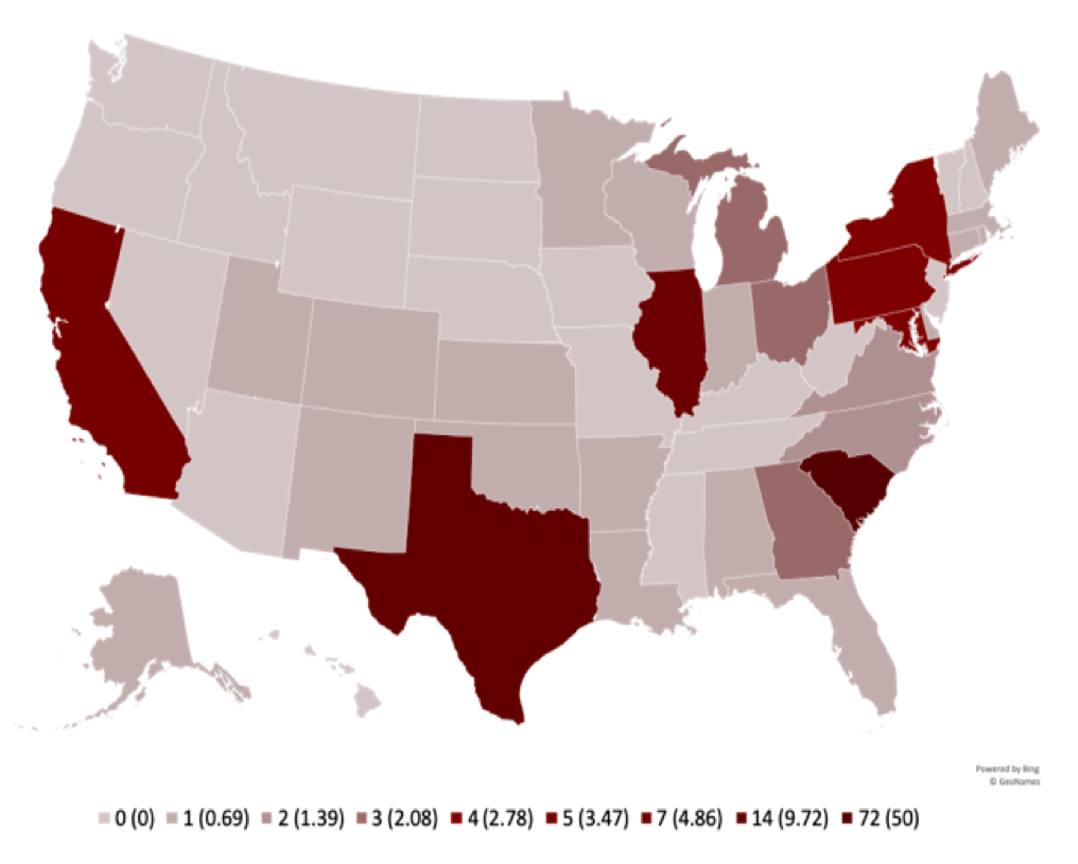

Supplement: S3 Appendix — Survey disseminated May-August 2019. Darker colors indicate greater numbers of survey participants. (TIF) [file pone.0252750.s003.tif]
