# Supplementary material for: Attitudes, beliefs and behaviors of religiosity, spirituality, and cultural competence in the medical profession: A cross-sectional survey study
Source: PLoS One. 2021 Jun 15;16(6):e0252750. doi: 10.1371/journal.pone.0252750 (PMC8205176; doi:10.1371/journal.pone.0252750)
Supplement: Attachment — Submitted filename: Response to reviewers 2-5-21.docx [file pone.0252750.s004.docx]

Response to Reviewers

Dear Rosemary Frey, Academic Editor, PLOS ONE,

Below are the comments from the reviewers and how we responded to their comments.

Thank you for this opportunity to address their concerns.

Sincerely,

Victoria Dillard, MS

Ann Blair Kennedy, LMT, BCTMB, DrPH

| Comment | Response |
| --- | --- |
| Editorial staff comments | |
| Please ensure that your manuscript meets PLOS ONE's style requirements, including those for file naming | Style requirements have been reviewed. Manuscript and files are in the correct format. |
| Please refer to any sample size calculations performed prior to participant recruitment. If these were not performed please justify the reasons. Please refer to our statistical reporting guidelines for assistance | For this preliminary study, sample sizes were not calculated. It was determined that post-hoc power analysis would be conducted to determine if the study was adequately powered. Additional information has been included in the text to clarify this situation.   - “As this study was seen as a preliminary exploration of differences between groups, sample size calculations were not performed prior to analysis; however post-hoc power analyses were conducted for any non-significant findings.” |
| Thank you for including your ethics statement:  "The xxxxxx Institutional Review Board approved this project (PRO#xxxxx). Participants were given a description of the survey and asked to “opt in” if they were willing to participate".  a. Please amend your current ethics statement to include the full name of the ethics committee/institutional review board(s) that approved your specific study.  b. Once you have amended this/these statement(s) in the Methods section of the manuscript, please add the same text to the “Ethics Statement” field of the submission form (via “Edit Submission”). | These have been completed. We have changed the text to read:   - “The University of South Carolina Institutional Review Board approved this project (Pro00087751).” |
| Please provide additional details regarding participant consent.  In the ethics statement in the Methods and online submission information, please ensure that you have specified what type you obtained (for instance, written or verbal, and if verbal, how it was documented and witnessed).  If your study included minors, state whether you obtained consent from parents or guardians.  If the need for consent was waived by the ethics committee, please include this information. | All participants were adults. Written consent to participate in the study was obtained from all participants.  The following was included in the manuscript for clarification of the informed consent process.   - “Participation in the study was voluntary and uncompensated. All participants were adults. Written consent to participate in the study was obtained from all participants.” |
| We note that you have stated that you will provide repository information for your data at acceptance. Should your manuscript be accepted for publication, we will hold it until you provide the relevant accession numbers or DOIs necessary to access your data. If you wish to make changes to your Data Availability statement, please describe these changes in your cover letter and we will update your Data Availability statement to reflect the information you provide. | We have submitted our data as a supplementary file that we understand will be uploaded to the figshare repository. |
| We note that Figure 2 in your submission contains map images which may be copyrighted.  All PLOS content is published under the Creative Commons Attribution License (CC BY 4.0), which means that the manuscript, images, and Supporting Information files will be freely available online, and any third party is permitted to access, download, copy, distribute, and use these materials in any way, even commercially, with proper attribution. For these reasons, we cannot publish previously copyrighted maps or satellite images created using proprietary data, such as Google software (Google Maps, Street View, and Earth)  We require you to either (a) present written permission from the copyright holder to publish these figure specifically under the CC BY 4.0 license, or (b) remove the figure from your submission | Figure 2 was created via excel. No images were copyrighted.  However, we determined that the information may be better displayed as “supporting information” instead and will be uploaded there.  “**S3 Appendix. Number of Survey Participants by State.**  Survey disseminated May-August 2019. Darker colors indicate greater numbers of survey participants.” |
| Reviewer 1 | |
| Background: Lines 82-90: Here you are describing how different religions hold different attitudes on same sex marriages – and then discuss immigration where an increase of immigrants has had a negative impact, but it’s not clear what the negative impact has been upon. (Apologies if I have missed this). The connection of this sentence to the need to deliver culturally appropriate care is not clearly demonstrated in the current text, as you explain what culturally competent care is after this sentence when it should come earlier. Your work would also benefit from a sentence or two discussing how religion and spirituality are an important component of culturally competent care. The background would benefit from discussing how religious and spiritual beliefs often intertwine with cultural beliefs, and so true cultural competency requires an understanding of the patient’s religion and spirituality. There are a few reviews, key textbooks that are worth mentioning all which discuss the role of a healthcare professionals religious and spiritual beliefs and the influence these can have during clinical encounters. It is well-established that if a healthcare professional does not have a religious or spiritual belief delivering spiritual care is difficult often awkward, different beliefs in relation to the patient can also be a barrier as they wish to not offend the patient. It is important to acknowledge that much of this work has been conducted on nursing professionals, specific diseases and healthcare conditions (e.g., cancer, palliative care, pain), if you are referring to a lack of evidence in specific medical situations then please clarify this. I have recommended some texts worth adding to this section: Shah, S., Frey, R., Shipman, K., Gardiner, F., & Milne, H. (2018). A survey to explore health care staff perceptions of spirituality and spiritual care working in a single district health area in New Zealand. European Journal of Integrative Medicine, 22, 1-9.  Chen, M. L., Chen, Y. H., Lin, L. C., & Chuang, L. L. (2020). Factors influencing the self‐perceived competencies in spiritual care of nurses in the long‐term care facilities. Journal of Nursing Management.  Delbridge, E., Taylor, J., & Hanson, C. (2014). Honoring the “spiritual” in biopsychosocial-spiritual health care: Medical family therapists on the front lines of graduate education, clinical practice, and research. In Medical family therapy (pp. 197-216).  Springer, Cham Donohue, P. K., Boss, R. D., Aucott, S. W., Keene, E. A., & Teague, P. (2010). The impact of neonatologists' religiosity and spirituality on health care delivery for high-risk neonates. Journal of palliative medicine, 13(10), 1219-1224.  Austin, P., Macleod, R., Siddall, P., McSherry, W., & Egan, R. (2017). Spiritual care training is needed for clinical and non-clinical staff to manage patients’ spiritual needs. Journal for the Study of Spirituality, 7(1), 50-63.  Best, M., Butow, P., & Olver, I. (2016). Why do we find it so hard to discuss spirituality? A qualitative exploration of attitudinal barriers. Journal of clinical medicine, 5(9), 77.  Best, M., Butow, P., & Olver, I. (2016). Doctors discussing religion and spirituality: A systematic literature review. Palliative Medicine, 30(4), 327-337. | We have repositioned the introduction, which included removing the portions on same sex marriage and immigration.  Thank you for offering the references. We have included the neonatology article as part of the introduction to demonstrate the role religion and spirituality may have in clinical decision-making. Please see lines 95-106.   - “When we consider the intersection of religion, spirituality, and culturally competent healthcare, one study found that among a group of neonatologists, there were no differences amongst religious and non-religious providers in the decision to withhold or withdraw life-saving treatment to neonates in the intensive care unit [10]. Nevertheless, 7% of the study participants reported no obligation to refer patients to an alternate provider for options that they would object [10]. This is further highlighted in a study done by Curlin et al, which demonstrated that 18% of U.S. physicians reported no moral obligation to refer a patient to an alternative provider willing to perform a procedure or offer a treatment that they feel is morally wrong [11]. This challenges one to consider how personal religious and spiritual beliefs may play a role in interactions with patients in the healthcare setting. As globalization continues and the population of the United States becomes increasingly diverse, the ability to care for diverse people is becoming ever more important for healthcare providers. Furthermore, the ability to do this despite one's own personal religious and spiritual belief systems reinforces an important angle of social and behavioral research.”   Further, our concluding paragraphs of the introduction acknowledge the robust literature that exists on the intersection of provider religion, spirituality, and the delivery of spiritual care in the clinical setting. The aim of our study was not to investigate spiritual care specifically, but to assess the impact provider religious and spiritual identities have on four core cultural competency domains: knowledge, skills and abilities, professional interactions, and systems-level interactions.   - “Although robust literature exists that explores provider religiosity and spirituality and the impact this may have on different aspects of culturally competent healthcare (specifically, spiritual care in clinical settings), to our knowledge, there have been no studies that have specifically assessed how providers’ religious and spiritual identities impact the domains of cross-cultural knowledge, culturally aware patient care skills and abilities, cross-cultural professional interactions, and healthcare systems-level interactions.”   The remainder of your recommendations can be found in the discussion portion of our manuscript. Specifically, under “Patient Care Skills & Abilities,” we have included the additional references to help explain our findings.   - “Possible barriers to discussing spirituality include insufficient time and training, confusion over differences between religion and spirituality, lack of vocabulary, invasion of privacy, and lack of clarity around the physician role in these discussions. Nevertheless, religious, spiritual, and cultural beliefs and practices are often interwoven, thus, cultural competency requires a thorough understanding of the patient’s religion and spirituality.” |
| The uniqueness of the research is not clear from the background – there have been plenty of studies that have explored health care professionals/physicians attitudes behaviours and beliefs in relation to religion and spirituality and the role these have in delivering spiritual care/culturally competent care. Equally there have been several reviews that have identified that cultural needs of patients are not addressed within clinical consultations with physicians, psychologists - we already have a wealth of studies exploring and identifying what’s missing – what gaps will your current study address having a clear aim and concluding statement about specific gaps you wish to address will benefit the background. | Thank you for your feedback. The study was not intended to evaluate providers’ ability to deliver spiritual care. The aim is to assess how providers’ spiritual and religious identities affect their ability to deliver culturally competent care in four specific domains as listed above. We hope we have provided some clarity as the specific gaps we are address by the following in the text:   - “Although robust literature exists that explores provider religiosity and spirituality and the impact this may have on different aspects of culturally competent healthcare (specifically, spiritual care in clinical settings), to our knowledge, there have been no studies that have specifically assessed how providers’ religious and spiritual identities impact the domains of cross-cultural knowledge, culturally aware patient care skills and abilities, cross-cultural professional interactions, and healthcare systems-level interactions.” |
| Methods: This section was very difficult to follow, may I suggest breaking it down in to the subheadings of the each of the scales that were used to build your survey, and under each sub-heading discussing the measure psychometrics and which items/domains you took for this survey. So something like: We developed our instrument on three questionnaires that have been used in healthcare service research the process will be described below: CCCQ – What the measure is, and how it used previously (with supporting references), what are its psychometrics, which questions you used in your survey, which items you amended and whether the psychometrics were impacted by the changes you made.  Follow the same format for each of the questionnaires (cultural linguistic checklist, CCSAQ) you used to build you survey then finish with how the survey was peer-reviewed by six experienced physicians and two medical students to provide stakeholders’ feedback on the question stems, responses, design, and flow of the questionnaire. | Thank you for your suggestions on reformatting this section. The methods section has been re-organized by survey heading. Under each heading is a description of the contents with the instruments used in each section as indicated. |
| How were participants identified? What sampling methods did you use? You used social media – which platforms did you use (e.g., twitter, facebook Instagram Linkedin?). | For further clarification in the paper we have included the following:   - “An online survey was selected to allow for a broader distribution of the survey across geographic areas and levels of training. Participants were recruited through social media (Facebook, Twitter) and local and national (ie. Student National Medical Association) email listservs.” |
| Was a sample size calculated – as you are assessing a lot of variables was this conducted when designing the original statistical analysis plan? If not why was this not conducted? | For this preliminary study, sample sizes were not calculated. It was determined that post-hoc power analysis would be conducted to determine if the study was adequately powered. Additional information has been included in the text to clarify this situation.   - “As this study was seen as a preliminary exploration of differences between groups, sample size calculations were not performed prior to analysis; however post-hoc power analyses were conducted for any non-significant findings.” - “Post-hoc power analyses were conducted using G*Power 3.1 software for any non-significant results.” - “The null results in the comparison between groups led us to conduct a post hoc power analysis to determine if our study was adequately powered. The priori analysis indicated that a power (1 - β) set at 0.80 and α = .05, two-tailed, sample sizes are suggested to include 67 participants in each group to detect medium effects (d=.5); large effects (d=.8) could be detected with only 27 participants in each group. However, the post hoc analysis to determine achieved power based upon means and standard deviations for each group yielded an effect size d = 0.117 with Power = 0.099.” |
| Results: Table 2 – not all of the data presented tallies up to N=144 (Location of practice, gender of the population, race/ethincity) this can be confusing to the reader – I am assuming the location of practice reflects that participants practiced at various locations, can this please be made clearer in the table layout. | Participants were permitted to select more than one option for location of practice and race/ethnicity.  Footnotes have been added to this table for clarification. |
| You haven’t discussed any regressions analyses, consider removing this from the analysis section, or add a section to the work to say that further exploratory analyses were not completed due to non-significant results | We have removed the multivariate analysis of variance and regression analysis from this section. This section now reads:   - “Quantitative data were analyzed using R statistical software (R Foundation for Statistical Computing, version 3.6.1) with alpha set at 0.05. Continuous variables are reported as mean ± standard deviation. Discrete variables are reported as N (%). Continuous variables were analyzed using Wilcoxon rank-sum test or two independent samples t-test which is two-tailed, while discrete variables were analyzed using Chi-square.” |
| Qualitative data collection was not mentioned in the methods, and but appear in the results but very little is added to the findings, either add a section around how qualitative data was also collected at the end of the survey through open-ended questions or remove this bit. If you intend to keep this bit, then please add the analysis you did (e.g., descriptive thematic analysis) and what were the main findings. | Upon review and your suggestion we have removed the qualitative results from the manuscript. We agree there was not much value added by keeping it included. |
| Discussion: Similar comments are noted to the results, there are several studies that can be used to compare findings to; please consider using some of the refs from earlier to discuss findings from your study. Truong, M., Paradies, Y., & Priest, N. (2014). Interventions to improve cultural competence in healthcare: a systematic review of reviews. BMC health services research, 14(1), 99. | Thank you for this additional reference - Several references were included specifically in the “Patient Care Skills & Abilities” section of the discussion. |
| This is an umbrella review making reference to a lot of useful papers that are worth considering in your discussion. There is limited discussion of the limitations – very little recommendations can be made from your paper as the sample is too homogeneous, the response rate was moderate – but as there is limited discussion of recruitment strategies, its hard to determine whether this was to be expected or not. Please consider adding a sentence or two on this. | We have included additional information on recruitment strategies in the limitations section.   - “Further, the recruitment platforms present their own limitations (Facebook, Twitter, and email listservs). The lack of diversity in the respondents may be a reflection of the social networks through which the survey was disseminated and regional bias. Different results may emerge with a more diverse sample.” |
| Reviewer 2 | |
| The manuscript is written and structured well, but has some areas that need improvement. |  |
| The main challenge with this study is the lack of statistically significant results, hence relying on indications from inferential statistics. This needs to be strengthened in the discussion to make up for the lack of the former. | We have added the following to help strengthen the discussion.  In the limitations section we have added:   - “Moreover, while a sample size of 54 total individuals would be required to see large effect sizes, the post hoc analysis yielded a power of ~0.1. Thus, we cannot rule out that there may be a difference between groups if sample sizes were larger.” |
| The abstract, under methods, needs more information - e.g. number of participants, analysis. | We have included additional information on recruitment strategies and analysis.  Abstract methods now reads:   - “Physicians, residents, and medical students were recruited through social platforms to complete an electronically delivered survey, gathering data regarding demographics, cultural competency, religiosity, and spirituality. Four composite variables were created to categorize cultural competency: Patient Care Knowledge, Patient Care Skills/Abilities, Professional Interactions, and Systems Level Interactions. Study participants (n=144) were grouped as Christian (n=95)/non-Christian (n=49) and highly religious (n=62)/not highly religious (n=82); each group received a score in the four categories. Wilcoxon rank sum and Chi-square tests were used for analysis of continuous and discrete variables.” |
| The introduction needs improvement. There is abundant literature on cultural competency and cultural humility in healthcare, and currently the text merely refers to little. | We have revised a significant portion of the introduction and have included additional references in the introduction and throughout the manuscript to enhance the context of the study. It is hoped that the revision of this section will be acceptable upon review. |
| Also, on page 3, the authors suggest that spirituality only emerged recently due to the shifting demographics presented. However, this is inaccurate and further literature on spirituality as a concept and spiritually-sensitive practice will help strengthen the contextual information of the study. | We have included additional language around spirituality as a concept.   - “According to a 2001 article written by the American Psychological Society, religion describes specific behavior patterns and practices that align with a traditional system of faith and worship, where spirituality involves the pursuit of an ultimate truth and reality that is sacred to an individual [5]. It is important to note the differences between these two concepts, as spirituality should not be confined to religion, but appreciated within a broader definition that encompasses meaning, purpose, and relationships [6].” |
| Equally, there is more literature regarding how clinicians take into account religion and spirituality when making decisions in practice. I would recommend to the authors to explore and re-position the context of the study. | The introduction has been amended to reflect the current literature. Please see the following:   - “When we consider the intersection of religion, spirituality, and culturally competent healthcare, one study found that among a group of neonatologists, there were no differences amongst religious and non-religious providers in the decision to withhold or withdraw life-saving treatment to neonates in the intensive care unit [10]. Nevertheless, 7% of the study participants reported no obligation to refer patients to an alternate provider for options that they would object [10]. This is further highlighted in a study done by Curlin et al, which demonstrated that 18% of U.S. physicians reported no moral obligation to refer a patient to an alternative provider willing to perform a procedure or offer a treatment that they feel is morally wrong [11]. This challenges one to consider how personal religious and spiritual beliefs may play a role in interactions with patients in the healthcare setting. As globalization continues and the population of the United States becomes increasingly diverse, the ability to care for diverse people is becoming ever more important for healthcare providers. Furthermore, the ability to do this despite one's own personal religious and spiritual belief systems reinforces an important angle of social and behavioral research.” |
| A justification of why the self-administered online survey was the best tool to meet the aims of the study would be helpful in appreciating the strengths of the method. | The following text was added:   - “An online survey was selected to allow for a broader distribution of the survey across geographic areas and levels of training.” |
| More information about the validity of the CCCQ and self-assessment checklist from the NCCC is necessary. | We have significantly reframed the section based upon suggestions of the reviewers within each section validity of the instruments are discussed. The large amount of revisions make it impracticable to paste all within the table. |
| The study divided participants in the four groups mentioned, but no significant results were produced. Were there other divisions attempted which may had been more telling? | Our study sought to determine whether there were differences between the scores of the groups. At this point in time we have not attempted other divisions, future analysis of different groups could be explored |
| On page 17 (first line), the text reads, '...were asked to describe...'. Yet, participants did not describe anything in their responses but a Likert style survey tool was used. | The text now reads:   - “Respondents reported their patient population by ethnicity, race, and religion as well as ranked their level of knowledge on various sociocultural and socioeconomic factors (health disparities, different healing traditions, bias, prejudice, and historical impacts of racism). “ |
| Instead of 'Buddhist', the religion, 'Buddhism' should be used in the text. | Thank you, this was corrected.   - “The three religions with the lowest response rate (respondents who were unable to answer) were Buddhism, Baha’i, and Sikhism. “ |
| It is unclear whether the researchers asked about sexuality or sex in the demographics. On pages 19-20, this is further confusing. | We did not ask our participants to tell us the sex or sexual identities of their patient population.  And have indicated this in the text:   - “Respondents were not asked to describe their patient population by sexual orientation, sexuality, sex or gender identity.” |
| At the end of the discussion, further analysis alluding to the transferability of the findings may be useful, especially as to make this relatable to other contexts and an international audience. | We have added the following in the discussion:   - “Our results in this particular section appear to align with several international studies on spirituality and spiritual care in the clinical setting. Specifically, in Taiwan, Australia, and New Zealand, there is a call for spiritual care education amongst both physicians and nurses in order to meet the growing needs of patients. Although our study took a broader approach to analyzing cultural competence, this alignment suggests a global need for enhanced cultural competency curriculum across multiple disciplines [23-25].” |
| In the limitations, the authors do not talk about the lack of statistically significant results. | We have added the following to enhance the limitations section:   - “Moreover, while a sample size of 54 total individuals would be required to see large effect sizes, the post hoc analysis yielded a power of ~0.1. Thus, we cannot rule out that there may be a difference between groups if sample sizes were larger.” |
| Figure 1 does not add anything but is redundant to the text that explains the process. | Will be placed in supporting information as **S2 Appendix**. |
| Figure 2 can be substituted with a couple of statements in the text - methods - that talk about participants. | Will be placed in supporting information as **S3 Appendix**. |
| Once again, this is an interesting study but needs to take into account abundant work undertaken in the field, to make a stronger claim for adding to the literature. | We hope we have addressed your concerns. Thank you. |
