# Supplementary material for: Attitudes, beliefs and behaviors of religiosity, spirituality, and cultural competence in the medical profession: A cross-sectional survey study
Source: PLoS One. 2021 Jun 15;16(6):e0252750. doi: 10.1371/journal.pone.0252750 (PMC8205176; doi:10.1371/journal.pone.0252750)
Supplement: Attachment — Submitted filename: Response to Reviewers .docx [file pone.0252750.s005.docx]

Response to Reviewers

Dear Rosemary Frey, Academic Editor, PLOS ONE,

Below are the second round of comments from the reviewers and how we responded.

Thank you for this opportunity to address their concerns.

We eagerly await your favorable response.

Sincerely,

Victoria Dillard, MS

Ann Blair Kennedy, LMT, BCTMB, DrPH

| Comment | Response |  |
| --- | --- | --- |
| Reviewer #1:  Thank you for resubmitting this. Thank you for suggesting the changes, but I am afraid the rationale for the study is still not quite clear or contextualised in the evidence-base. There are many aspects to culturally competent care as you have identified, why have you chosen specifically on religion/spirituality? Why not look at cultural knowledge? As religion and spirituality is not the only aspect of cross-cultural care? What about clinicians overcoming language barriers, cultural beliefs that are not related to religion/spirituality?  I will reiterate that the novelty of your study is still not clear as there are ample reviews around delivering and supporting implementation of culturally competent care in various clinical contexts (e.g., cancer, mental health), which has looked at systems level approach also (i.e., clinician characteristics as barriers/facilitators, organisations as barriers/facilitators, clinical training barriers/facilitators and patient characteristics). How is your study different there is a clear enough rationale why exploring physicians RS would impact culturally appropriate care - why has this been given importance in delivering culturally competent care? The two studies you cite are not enough to support this as culturally competent care is more than patients religious and spiritual beliefs. | Thank you for this comment. The focus of this study centered on how physician religiosity and spirituality intersect with certain aspects of culturally competent healthcare. This was our research question and the direction we were interested in. We believe the other factors you mentioned are important as well, and our survey questions do reflect many of them, including knowledge, language, and cultural beliefs (please see Table 1).  For context, the idea of the study branched from a personal experience of one of the authors. Growing up in South Carolina, the Christian religion is a part of the fabric of life. Unfortunately, from personal observation, religion in the South can be weaponized to marginalize some populations. This manifests as implicit and sometimes explicit biases in the clinical setting and beyond. Because of these experiences, one of our authors believes they developed their own implicit bias toward highly religious peoples. For example, when someone would say, “I am Christian,” this author would hold their breath, tighten up, and wait for something negative to be said. This happened in a classroom setting and in another location. These encounters led to an inquiry about how religion may impact cultural competency. Subsequently, our literature search found no studies that explicitly researched this topic according to the domains we have selected. By dividing our participants into highly religious/majority religion versus not highly religious, we believe this was the most appropriate way to analyze the different groups.  For clarity, the focus of our study was not on patient religiosity and spirituality, but more so on physician R/S and how this may impact the diverse patient populations we serve. Further, it was simply looking to see if these two different groups had different levels of cultural competence. |  |
| In addition you have created a measure for exploring personal values (this was exploring intrinsic and extrinsic religiosity) there are validated measures for exploring these variables you measured e.g., DUREL for extrinsic religiosity, Religious orientation scale Intrinsic religiosity im not entirely sure why this was the case? this further limits the validity of your findings as this survey item has not been tested for validity and reliability. | Thank you for this comment. There was an error in this particular section when discussing how these questions were developed. They were based on the Pew Research Center’s Religious Landscape Study. <https://www.pewforum.org/2015/11/03/u-s-public-becoming-less-religious/>  This has been corrected and now reads:   - “These questions were based on the Pew Research Center’s Religiosity Landscape Study [1].”   Knowing that the remainder of the survey had many questions and in an attempt reduce respondent burden, we determined that the Pew Research Questions about religiosity would provide enough rigor. While there might not be reliability and validity statistics for these few questions, there is a rigorous methodology for the development of these questions by Pew.  Here’s a website <https://www.pewresearch.org/methods/u-s-survey-research/questionnaire-design/>  We have also addressed this further in the limitations portion of the manuscript.   - “Our study is further limited by the absence of a validated tool like the DUREL (The Duke University Religious Index) to measure religious involvement.” |  |
| I'm not entirely sure why the analysis was ran in R now instead of SPSS? Have you re-ran the analysis? | Our original IRB application states that the analysis was to be performed in SPSS. When we brought on another investigator to help with the analysis, we neglected to clarify which package they used. It was in the specific revisions we realized the use of R now instead of SPSS. |  |
| Reviewer #2:  Many thanks for submitting the revised manuscript. The re-structuring, clarity of information and enhancement of content have certainly improved the submission. I would like to offer that some further proofreading will help tidy up very few typos. While the cross-reference to the Figure 2 (in the text) is deleted (as this is moved to supplementary documents), the sentence still ends with 'within' but is incomplete (no page number in the revised document). | Thank you so much for your feedback.  This sentence reads:   - “Characteristics of the 144 participants who completed the survey are shown in Table 2 and S3 Appendix.”   The remainder of the document has been proofread and corrections made as needed. |  |
| I would also encourage the authors to triple check all references matching the list at the end and citation style. In the text, the name Austin appears, for example, as an in-text citation, instead of the number assigned to the reference in the list at the end. | All references and citations have been reviewed and corrected where needed.  Thank you. | |
